# Application of Rotation Rate Sensors in Modal and Vibration Analyses of Reinforced Concrete Beams

**DOI:** 10.3390/s20174711

**Published:** 2020-08-20

**Authors:** Piotr Adam Bońkowski, Piotr Bobra, Zbigniew Zembaty, Bronisław Jędraszak

**Affiliations:** Faculty of Civil Engineering and Architecture, Opole University of Technology, ul. Prószkowska 76, 45-758 Opole, Poland; p.bonkowski@po.edu.pl (P.A.B.); p.bobra@po.edu.pl (P.B.); b.jedraszak@po.edu.pl (B.J.)

**Keywords:** modal analysis, rotational mode, gyroscope, rotation rate sensors, UHPC, reinforced concrete beams, vibration

## Abstract

The recent rapid development of rotation rate sensor technology opens new opportunities for their application in more and more fields. In this paper, the potential of rotational sensors for the modal analysis of full-scale civil engineering structural elements is experimentally examined. For this purpose, vibrations of two 6-m long beams made of ultra-high performance concrete (UHPC) were measured using microelectromechanical system (MEMS) rotation rate sensors. The beams were excited to vibrations using an impact hammer and a dynamic vibration exciter. The results of the experiment show that by using rotation rate sensors, one can directly obtain derivatives of mode shapes and deflection shapes. These derivatives of mode shapes, often called “rotational modes”, bring more information regarding possible local stiffness variations than the traditional transversal and deflection mode shapes, so their extraction during structural health monitoring is particularly useful. Previously, the rotational modes could only be obtained indirectly (e.g., by central difference approximation). Here, with the application of rotation rate sensors, one can obtain rotational modes and deflection shapes with a higher precision. Furthermore, the average strain rate and dynamic strain were acquired using the rotation rate sensors. The laboratory experiments demonstrated that rotation rate sensors were matured enough to be used in the monitoring and modal analyses of full-scale civil engineering elements (e.g., reinforced concrete beams).

## 1. Introduction

Rotational sensors (gyroscopes) measuring rotational velocity are finding their application in more and more fields. To name only a few, one can mention seismology [1,2,3], structural health monitoring (SHM) [4,5,6], military [7], automotive [8], or in posture control [9,10]. In civil engineering, microelectromechanical system (MEMS) gyroscopes are getting particular attention because of their miniaturization and lower cost compared with the other systems [11]. These properties may be especially useful in the SHM of building structures. These structures are usually complex, with many places where sensors measuring strain are difficult or even impossible to be mounted on, e.g., on a surface with many microcracks. The application of rotation measurements in SHM started with numerical simulations [12,13,14] and was followed by experiments [4,6,14,15,16].

An important part of the information on structural behavior is knowledge about the changes in mode shapes following damage development. To achieve this goal, mode shape extraction methods where developed [13,17,18,19,20,21,22]. It is well known that the effects of damage are better reflected in the modal spatial derivatives than in the modes themselves. For these reasons, derivatives have been the subject of intensive research [13,17,20,23,24]. Following a nomenclature from previous studies, the spatial derivatives of transversal mode shapes are called rotational mode shapes [13,23] and curvature mode shapes [17].

**Figure 1:**
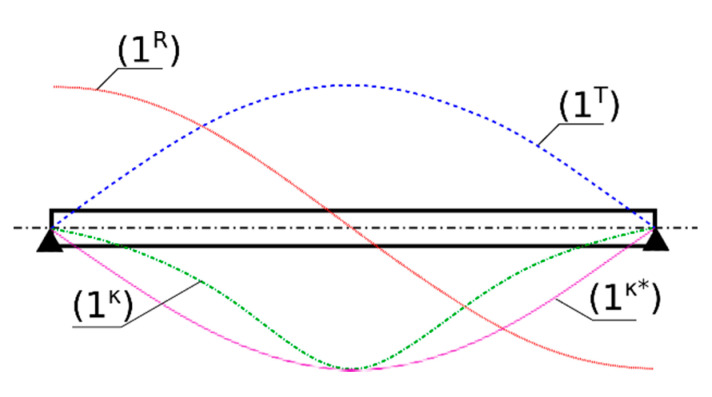
Simply supported Euler–Bernoulli beam and its (1^T^) first transversal natural mode, (1^R^) first rotational natural mode (slope of the first transversal mode), (1^κ^) first curvature natural mode, and (1^κ^*) approximation of the first curvature natural mode as a second derivative of the first transversal mode.

Consider the transversal vibrations, *w(x,t)*, of a simply supported Euler–Bernoulli beam under arbitrary excitations, slope of the beam axis r(x,t), and respective curvature κ(x,t). Solving the eigenproblem of the beam, one can obtain its natural frequencies and modes. In Figure 1, the first natural mode of the beam is plotted and denoted as 1^T^, where superscript T denotes the transversal character of the first natural mode of this beam. Calculating the first spatial derivative of the transversal mode shape $r=w^{\prime }$ with respect to *x* (slope), one obtains so called first rotational mode shape (1^R^), representing the rotations of the beam axis along the first mode. Analogously, one can obtain the so-called “curvature” mode (1^κ^), which describes the changes of the curvature along the first mode. The curvature of the beam can be obtained from its transversal displacements, *w = w(x,t)*, by the familiar formula of the structural mechanics, as follows:

(1) $$\kappa =\frac{w^{\prime \prime }}{{\left(1+{w^{\prime }}^{2}\right)}^{\frac{2}{3}}}$$

which also holds for the natural modes. However, typically, the curvature mode shape is approximated by second derivative of the transversal mode shape, $\kappa \left(x\right)\approx w^{\prime \prime }\left(x\right)$ (1^κ^* mode). The smaller the slope, *r*, the closer the approximate curvature, $\kappa^{*}$, to the actual curvature, κ. In fact, for typical deflections of civil engineering structures, the curvature can be successfully obtained using the $\kappa^{*}$ approximation.

Because of the earlier lack of effective rotational sensors, indirect methods were used to obtain the rotational and curvature mode shapes. For this purpose, measurements from acceleration sensors were used as the inputs for the central difference approximation of the transversal mode shapes. The rotational modes can be obtained from the translational modes, using the following approximation:

(2) $$r_{i}^{n}=\frac{-w_{i-1}^{n}+w_{i+1}^{n}}{2h},$$

where $w_{i}^{n}$ is the transversal modal displacement of the *n*-th mode at the *i*-th point, as measured by the translational sensors. The curvature can be approximated by the second central difference, as follows:

(3) $$\kappa_{i}^{n}=\frac{w_{i-1}^{n}-2w_{i}^{n}+w_{i+1}^{n}}{h^{2}},$$

The drawbacks of using translational sensors and finite difference formulas are particularly pronounced in the areas of the beam where translations are smaller, while rotations are higher, e.g., near the supports or ends of the beam. The problem is magnified in cases of the presence of large measurement noise, which is a typical situation when recording the vibrations of reinforced concrete beams.

Other techniques can also be utilized, e.g., laser measurement techniques [25] or strain-based methods [23]. Recently, Sung et al. [14] undertook the challenge of applying rotation rate sensors in flexibility-based SHMs, including elements of modal analysis, without, however, further mode derivative analyses. The paper includes numerical simulations and small-scale laboratory tests. They pointed out some advantages of rotation rate measurements in SHM, but also expressed scepticism in their wider technical usage due to alleged excessive noise in the vibration measurements in real technical applications.

A similar area to the analysis of rotation and curvature mode shapes is the utilization of rotation sensors in the slope analyses of beams under flexural vibrations. In recent years, researchers started to report particular advantages of rotation measurements for SHM purposes. Kokot and Zembaty [12] found that rotation measurements can enhance the stiffness “reconstruction” of beams and frames. Al-Jailawi and Rahtamala [6,16] studied gyroscope sensors in transmissibility analyses, and found that rotation rate sensors are particularly sensitive to damage localization. Zembaty et al. [4] experimentally studied the application of gyroscopes in the vibration measurements of plexiglass beam indirect strain sensing. Huras et al. [26] found that rotation measurements are potentially applicable in plastic hinge monitoring in seismically induced frame structures. Huseynov et al. [27] successfully used rotation measurements for the SHM purposes of bridge structures.

It is interesting to note that the aforementioned displacement derivatives can also be measured by an alternate technique of digital shearography [28,29]. For example, Yang et al. obtained the slope displacement field of turbine blades using shearography [30], while Katunin et al. [31] experimentally obtained modal rotation fields of a small aluminium plate and used it for damage detection in combination with wavelet analyses. Shearography has also been successfully used in obtaining second-order displacement derivatives [32,33]. However, rotation rate sensing can be a better choice in the areas where shearography has shortcomings, e.g., for the large rigid body motion included in the vibrations [30] or for structural elements where optical measurements are not possible.

The purpose of this paper is to examine the possibility and the advantages of using modern gyroscopes in

- direct extraction of the rotational modes (spatial derivatives of translational modes),
- direct slope measurements (rotational deflection shape) during vibration measurements with an inertial exciter,
- average strain control during vibrations of cracked reinforced concrete rods.

For this purpose, full scale, 6-m long, ultra-high performance concrete (UHPC) beams were measured in intact and partly damaged states in laboratory conditions. The monitoring of vibrations of concrete and UHPC beams brings particular challenges to the field of SHM (see, e.g., [34,35,36,37,38,39]). For these reasons, the application of rotation rate sensors in the modal extraction and vibration analyses of these beams can bring particular advantages. Particular attention is paid to compare slopes derived directly, using rotation measurements and by using derivatives of translational measurements.

## 2. Materials and Methods

### 2.1. General Description of the Analysed Beams

**Figure 2:**
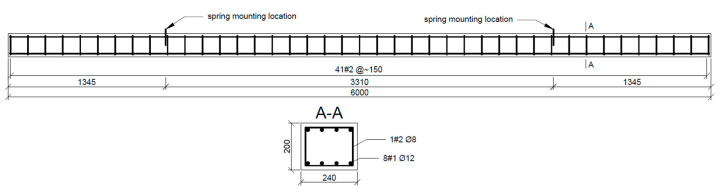
Geometry of the analysed beams (dimensions in mm).

In this research, the experiment was conducted on two UHPC beams, as shown in Figure 2, denoted as beam UHPC1_L and beam UHPC2_L. The beams were reinforced using traditional rebars and with distributed reinforcement (fibers), following the specific requirements for the UHPC mixture. In each of beam, a different fiber type and concrete mix was used, which resulted in different mechanical properties, as shown in Table 1.

**Figure 3:**
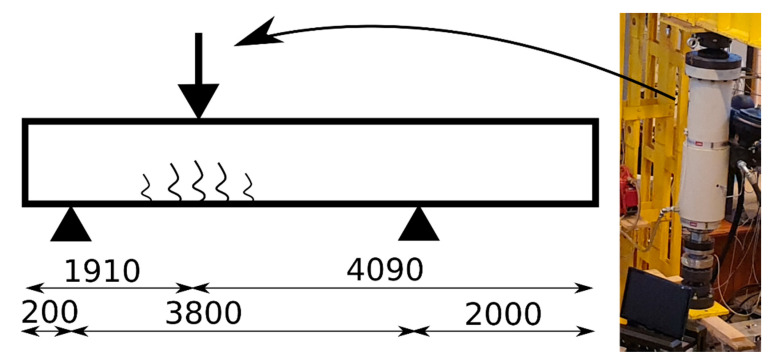
A sketch showing the infliction of damage to the beams using an Instron actuator (photograph on the right). Dimensions in mm.

The main aim of the reported research was to carry out modal extraction from intact and partly damaged beams. First, the beams were tested in intact states, and next, the damage was inflicted to the beams supported at their ends using a heavy Instron actuator (Instron, Norwood, MA, USA). The actuator and supports of the beams were located asymmetrically in order to examine the changes in the mode shapes introduced by the non-uniform damages of the beams (Figure 3).

**Figure 4:**
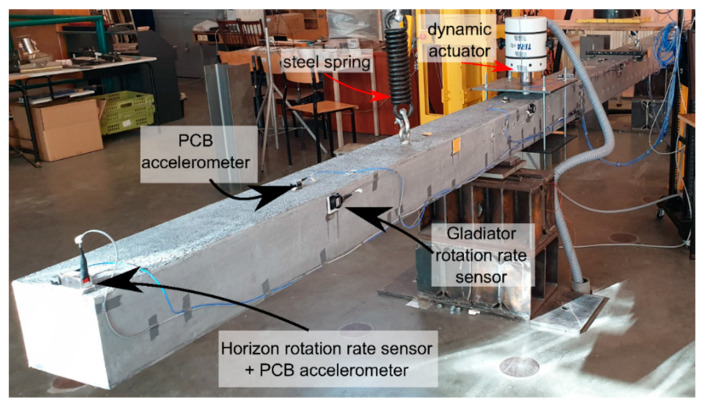
Experimental set-up for the tested beams.

After inflicting the damages, the Instron actuator was removed, and the beam was released from being supported to hanging in the “free–free” boundary conditions for the modal tests. The free–free conditions were chosen for the diagnostic experimental phase in order to compensate for any distortions coming from the supports. This was achieved by hanging the beams on two steel springs located in the individual nodes of their first free–free vibration modes (Figure 4).

**Figure 5:**
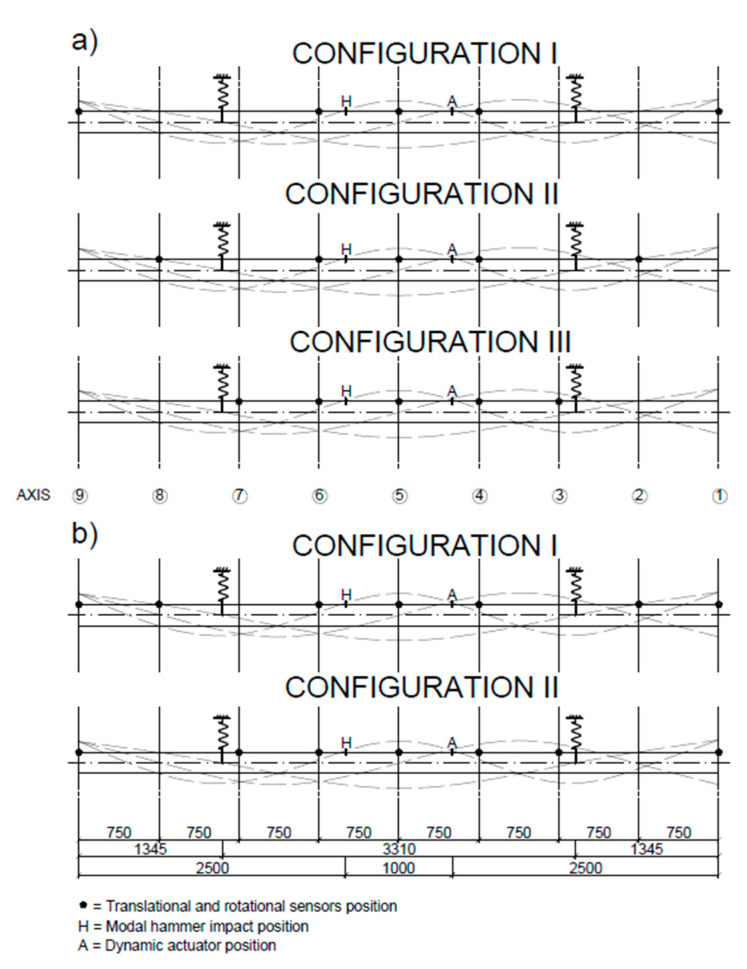
Sensors configuration for (**a**) beam UHPC1_L and (**b**) beam UHPC2_L. Dashed lines show the theoretical mode shapes computed for the Euler–Bernoulli beam.

The vibrations of beams were measured using PCB 3711E1110G and PCB 3711B1110G MEMS accelerometers (PCB Piezotronics, Depew, NY, USA), which were collocated with rotation rate sensors. For this purpose, the Horizon (Systron Donner Inertial, Concord, CA, USA) and Gladiator (Gladiator Technologies, Snoqualmie, WA, USA) gyro sensors were applied (Table 2). The respective sensors were placed at axes located with a 0.75-m spacing. Measurements were conducted using a 12 channel Data Translation DT9837 (Data Translation, Marlborough, MA, USA) acquisition system. In order to obtain the precise vibration characteristics of the beams, the measurements were divided into three (beam UHPC1_L) or two (beam UHPC2_L) stages, where the outmost Gladiator sensors roved along the beam length, as shown in Figure 5. In each sensor configuration, there were five measurement points for beam UHPC1_L, and seven measurement points for beam UHPC2_L. This was done to minimize the number of simultaneously working data channels, limited here to 12. After connecting the data from each configuration, nine measurement points were obtained in every beam in total. The rotational sensor positions are shown in Table 3.

The beams were excited using a PCB 086D20 (PCB Piezotronics, Depew, NY, USA) modal hammer (impact hammer test) and with the Tira Inertial Vibration Test System TV 51165-IN (TIRA GmbH, Germany, EU) (harmonic excitations test).

### 2.2. Modal Parameter Extraction Using Impact Hammer

**Figure 6:**
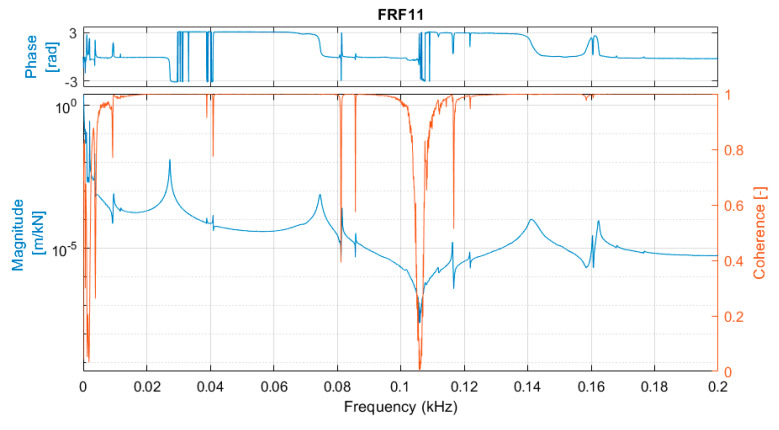
The exemplary frequency response functions (FRFs) of the analyzed beam UHPC2_L in configuration 1.

There are many techniques for modal extraction in operational and experimental modal analyses (see, e.g., [40,41,42]). In this research, the functions implemented in the MATLAB (Mathworks, Natick, MA, USA) Signal Processing Toolbox were utilised. Briefly, the procedure was as follows: First, the translational acceleration, rotation rate, and hammer force measurements were recorded during the impact test in free–free conditions (beams hanging on steel springs, as shown in Figure 4). Then, frequency response functions (FRFs) were obtained using the $H_{1}$ estimator [41], as follows:

(4) $$H\left(f\right)=\frac{G_{yx}\left(f\right)}{G_{xx}\left(f\right)},$$

where $G_{yx}\left(f\right)$ is the single-sided cross spectral density of input *x* and output *y*, and $G_{xx}\left(f\right)$ is the single-sided auto spectral density of the input. The FRFs were calculated separately for the translational accelerations and rotational rates. The example FRF is shown in Figure 6.

**Figure 7:**
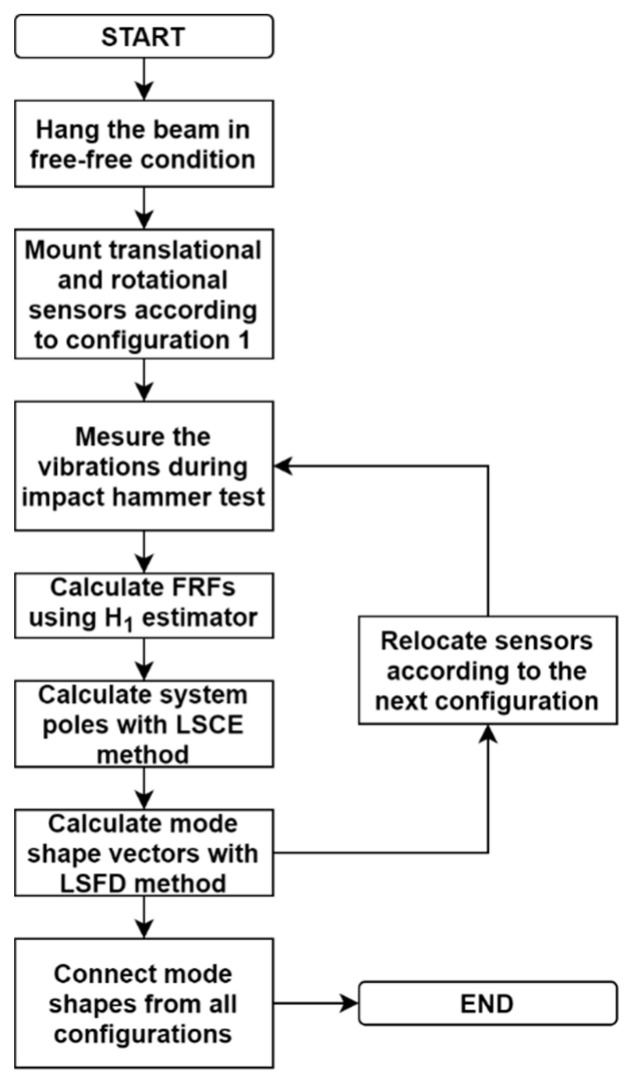
Flowchart of the procedure of the modal parameter extraction using an impact hammer.

Next, the system poles were extracted using FRFs and the least squares complex exponential (LSCE) method [43]. The LSCE method is an upgrade of the historical 1795 Prony’s method [44], which allows for including multiple impulse response functions. The system poles were used to calculate the natural frequencies and damping coefficients. Finally, the mode shape vectors were estimated using the least squares frequency domain (LSFD) method by solving the following equation:

(5) $$\left[\Lambda \right]\left\{A_{p}\right\}=\left\{H_{pq}\left(\omega \right)\right\},$$

where [Λ] is the eigenvalue matrix, $\left\{A_{p}\right\}$ is the residue coefficient vector, and $\left\{H_{pq}\left(\omega \right)\right\}$ is the frequency response. More details about the procedure can be found in the literature [41,45]. The procedure was repeated for sensor configurations II and III in order to better refine the mode shapes. The flowchart of this procedure is presented in Figure 7.

### 2.3. Inertial Vibration Exciter Tests

The beams were additionally tested using a Tira inertial vibration exciter with a harmonic excitation signal near resonant frequencies. During the tests, the translational accelerations and rotation rates were measured in configurations I–III. The translational and rotational steady-state deflection shapes were estimated as the square root of the peaks in the cross spectral density of the input signal, as follows:

(6) $$d^{n}=\max\left(\sqrt{G_{xx}^{n}\left(f\right)}\right),$$

$d^{n}$ is the steady state translational or rotational deflection shape in node *n*.

### 2.4. Strain Measurements Using Rotation Rate Sensors

During testing with the inertial vibration exciter, an average strain between the measurement axes (Figure 5 and Table 3) was acquired by two rotation rate sensors. The strains were obtained following approximate relations between the rotation rate and strain rate [4], briefly repeated below. In particular, the relation between the curvature and strain at the beam surface equals the following:

(7) $$\kappa =\frac{d\vartheta }{dx}=\frac{d^{2}w}{dx^{2}}\approx \pm \frac{2\varepsilon_{max}}{h}\;$$

where κ is the curvature, ϑ is the axis rotation, h is the height of the beam, and $\varepsilon_{max}$ is the maximum strain at the beam surface given by following formula

(8) $$\varepsilon_{max}=\pm \frac{hd\vartheta }{2dx}\;$$

After selecting the finite distance, Δx, between the respective two rotation rate sensors, and taking into account the fact that the rotation rate, $\dot{\vartheta }$, is the measured quantity, the strain rate and strain could be obtained with the following equations:

(9) $${\dot{\varepsilon }}_{max}\left(t\right)=\pm \frac{h\;\Delta \dot{\vartheta }\left(t\right)}{2\Delta x}\;$$

(10) $$\varepsilon_{max}\left(t\right)=\pm \int \frac{h\;\Delta \dot{\vartheta }\left(t\right)}{2\Delta x}dt$$

### 2.5. Comparing the Modes

The dynamic modes measured during the diagnostic experiments were compared with each other using the familiar modal assurance criterion (MAC) [41,46]:

(11) $$MAC_{rs}=\frac{{\left|{\left\{\psi \right\}}_{r}^{Tr}{\left\{\psi \right\}}_{s}\right|}^{2}}{\left({\left\{\psi \right\}}_{r}^{Tr}{\left\{\psi \right\}}_{r}\right)\left({\left\{\psi \right\}}_{s}^{Tr}{\left\{\psi \right\}}_{s}\right)}$$

where {ψ} is the mode shape vector, and ${\left\{\psi \right\}}^{Tr}$ is the transpose of the mode shape vector. The closer to one the MAC value, the more similar two mode shape vectors are.

## 3. Results

The tests began from the modal analyses of the two beams in an “intact” state, using the impact hammer and LSCE method to extract the first three vibration modes of the beams. The extracted modal parameters are shown in Table 4. The damping ratio is similar for the two beams. Beam UHPC2_L, despite having a larger mass, also has higher natural frequencies because of its greater stiffness.

**Figure 8:**
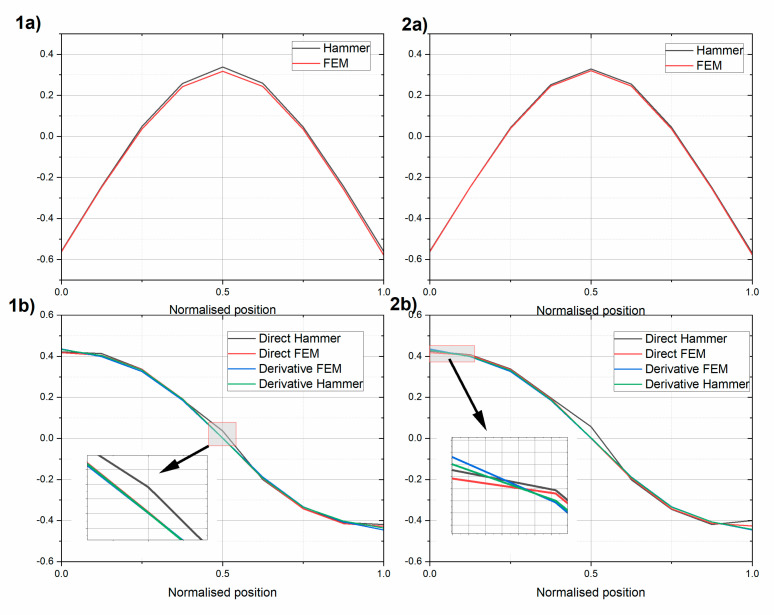
Comparison of the first (**a**) translational and (**b**) rotational mode of (1) beam UHPC1_L and (2) beam UHPC2_L. Results for the intact beams.

**Figure 9:**
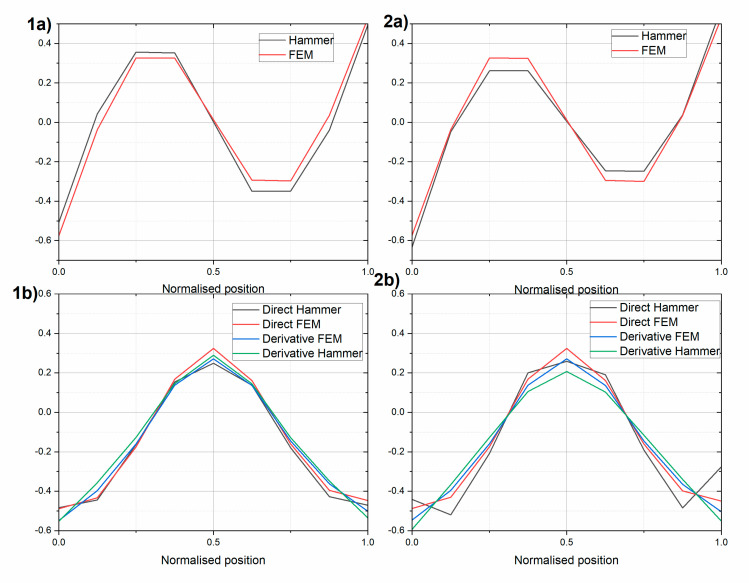
Comparison of the second (**a**) translational and (**b**) rotational modes of (1) beam UHPC1_L and (2) beam UHPC2_L. Results for the intact beams.

Next, the first two mode shapes were extracted for the two beams (Figure 8 and Figure 9, respectively). The experimental mode shapes were compared with the mode shapes obtained using the finite element model (FEM) in SAP2000 (Computers and Structures, Walnut Creek, CA, USA) software [47]. In the FEM, the UHPC beams were modelled by “beam” finite elements, which account for bending and shear deformations, using the lumped matrix formulation. The beams were modelled with 61 elements. The maximum element size was 0.1 m. The unified elastic material properties were assumed using $\rho =2400\;\mathrm{kg}/m^{3}$, E=43.75 GPa, and ν=0.2 for beam UHPC1_L, and $\rho =2725\;\mathrm{kg}/m^{3}$, E=65.35 GPa, and ν=0.2 for beam UHPC2_L. The experimental free–free condition was reproduced using spring boundary FEM elements with values and locations equal to the ones applied in the laboratory. The FEMs included masses from additional equipment, i.e., an Instron actuator fixture (10.8 kg) and inertial exciter (39.6 kg). In Figure 8 and Figure 9, additionally, central difference approximation (Equation (1)) of the rotational modes using measured and FEM translational modes are shown. The translational and rotational mode shapes were normalized using the Euclidean norm, as follows:

(12) $$v^{norm}=\frac{v}{\left|\left|v\right|\right|}=\frac{v}{\sqrt{\sum_{k=1}^{n}{\left|v_{k}\right|}^{2}}}\;$$

where $v^{norm}$ is the either normalised translational or rotational mode shape vector; v is the either translational or rotational mode shape vector using FEM, experimental modal analysis, or a derivative of the respective translational mode shape; and $v_{k}$ is the value of the mode shape vector at the *k*-th node.

The experimental translational and rotational first mode was in very good agreement with its FEM model. The MAC values for the translational mode and rotational modes differed by less than 1% for the two beams (see Table 5 and Table 6). The rotational mode obtained with the modal hammer technique indicates that there was a nonzero rotation in the middle of the beam. This experiment was also repeated using an inertial vibration exciter. The rotational modes obtained using the central difference approximation were close to the modes obtained directly, yet some discrepancies were observed, especially at the end of the beams. They appeared even if the purely numerical FEM translational mode was used.

The experimental and numerical second mode shapes were still similar, but with slightly lower MAC values. The translational MAC values were over 0.97, and the rotational values are shown in Table 7 and Table 8. The discrepancies between the directly obtained rotational modes and those using the central difference approximation were even more visible. The second rotational modes obtained using gyro sensors differed somewhat from what was expected using the FEM analysis. This was probably because two different gyro sensors were used, and the bandwidth of the Gladiator sensor was equal 200 Hz and of the Horizon was equal 60 Hz, while the second natural frequency was above 65 Hz. Table 3 shows a comparison of the gyro sensor positions.

**Figure 10:**
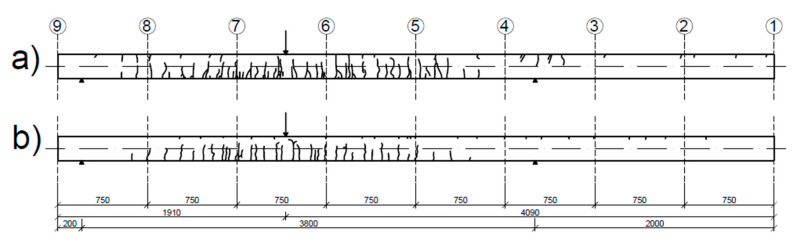
Sketch of cracks in (**a**) UHPC1_L after the test procedure and (**b**) UHPC2_L after the test procedure.

After inflicting damage to the beams, the translational and rotational modes were extracted with the same technique. In Figure 10, sketches of the crack distribution in the beams after a damaging load stage are shown. In the damaging load stage, plastic hinges started to develop in the beams, and the maximum inflicted load was equal to 49.8 kN for the UHPC1_L beam and 65.2 kN for the UHPC2_L beam. The modal parameters obtained in the damaged state are shown in Table 9.

**Figure 11:**
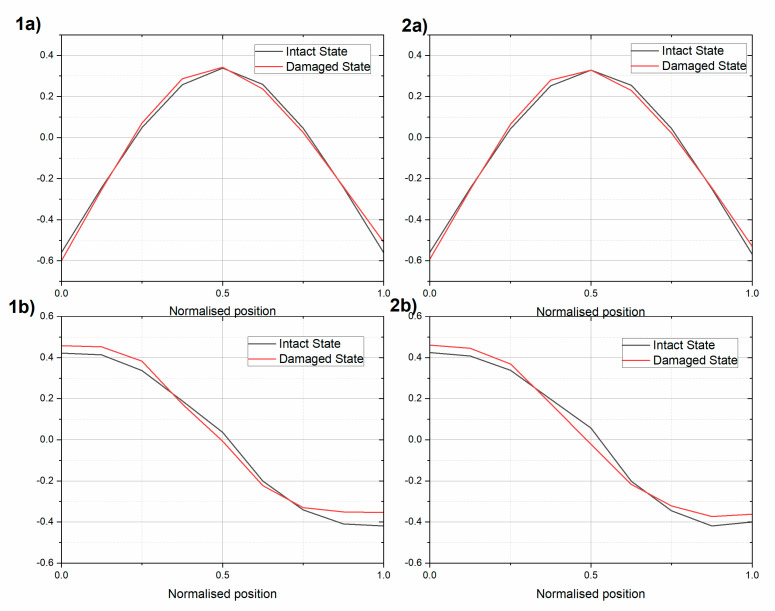
Comparison of the first (**a**) translational and (**b**) rotational modes of the intact and damaged (1) beam UHPC1_L and (2) beam UHPC2_L.

In Figure 11, a comparison of the first modes between the intact and damaged beams is presented. The difference in the natural modes between the damaged and intact state was more pronounced for the rotational mode than for the translational mode. The MAC value between the intact and damaged states equaled 0.9932 and 0.9949 for the translational mode of UHPC1_L and UHPC2_L, respectively. For the rotational mode, the MAC value equaled 0.9847 and 0.9854 for the UHPC1_L and UHPC2_L beams, respectively.

**Figure 12:**
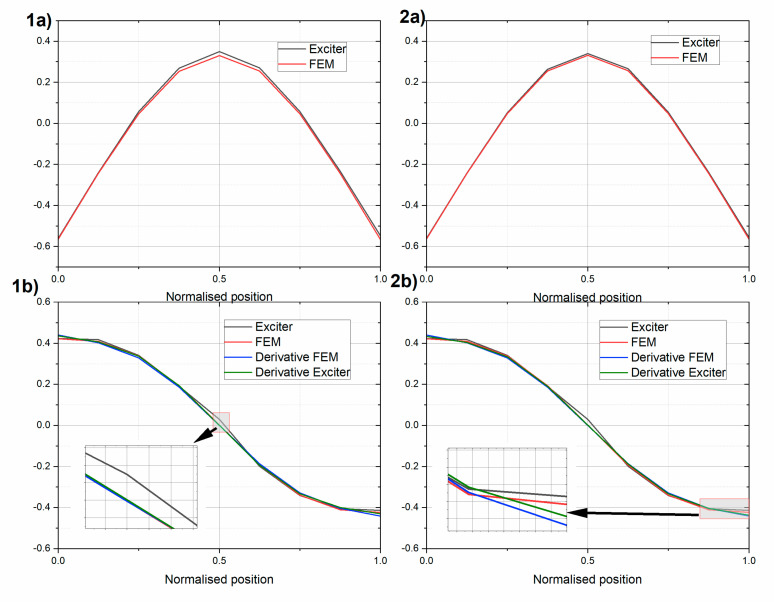
Amplitudes of (**a**) translational acceleration and (**b**) rotation rate of (1) beam UHPC1_L and (2) beam UHPC2_L during the inertial vibration exciter tests. Results for the intact beams.

The deflection shapes were also extracted using an inertial vibration exciter. The results for the actuator working near the first resonant frequency are shown in Figure 12. The presented results are normalized according to Equation (12). The experimental results were compared using FEM steady state response analysis. The overall results were similar to the ones obtained using the modal hammer.

**Figure 13:**
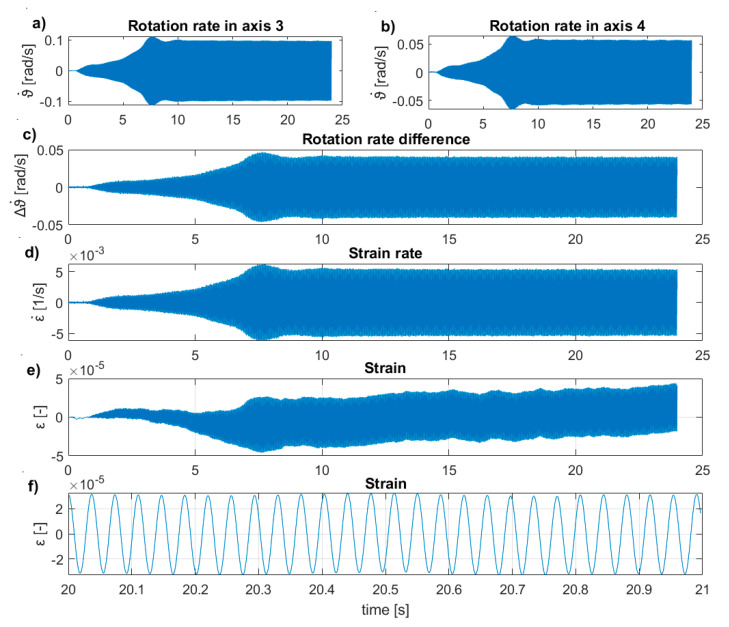
Result for the intact UHPC2_L beam induced by the inertial vibration exciter with 27.26 Hz. (**a**) Rotation rate at axis three, (**b**) rotation rate at the axis, (**c**) difference in rotation rate between axis three and axis four, (**d**) average strain rate between axis three and axis four, (**e**) average strains between axis three and axis four, and (**f**) short time window of average strains between axis three and axis four.

During the tests with the inertial vibration exciter, the feasibility for measuring the average strain rates and the strains using two rotation rates sensors according to the procedure presented in Section 2.4 was analyzed. The presented results are for the UHPC2_L beam in a free–free condition. The sensors and inertial exciter were set according to the configuration II scheme shown in Figure 5. The average strain rates and strains were measured for the area between axis three and axis four using the Gladiator sensors. The exciter worked with 27.26 Hz. First, the rotation rates in the selected axes were measured (Figure 13a,b), which allowed for calculating the rotation rate difference between the two sensors, $∆\dot{\vartheta }={\dot{\vartheta }}_{3}-{\dot{\vartheta }}_{4}$ (Figure 13c). Next, the strain rate, ${\dot{\varepsilon }}_{max}$, was calculated using Equation (9), with a beam height of h=0.20 m, and a distance of Δx=0.75 m between the sensors (Figure 13d). The strain rates were integrated according to Equation (10) (Figure 13e). Such operation resulted in the strains randomly drifting from a zero value, because of, e.g., sensor noise. In this case, the measurements could be corrected by using, e.g., a high-pass filter. Here, we restricted the analysis to a short time window and used a linear detrend function of $\varepsilon_{corrected\;}\left(t=20–21\;s\right)=detrend\;\varepsilon \;\left(t=20–21\;s\right)$ (Figure 13f). As such, the procedure allows for control of the average strains during the vibration measurements.

## 4. Discussion and Conclusions

Mode extraction techniques are as old as modal analysis. In the early days, only translational modes could be directly acquired. The mode derivatives, e.g., rotational modes, could be measured only indirectly, because of the insufficient accuracy or lack of miniaturization of the rotational sensors. With the recent rapid development of the MEMS rotation rate sensors, the capacity of the modal analysis techniques can be expanded to directly obtained derivatives, e.g., the rotational modes needed in SHM.

In this paper, the experimental results of the application of the rotation rate sensors in the modal analysis of two 6-m UHPC beams are reported. The first three natural frequencies of the analysed elements ranged from 19.40 to 140.66 Hz. The results show that the rotation rate sensors can be applied with success in the modal extraction of full-scale civil engineering structures. Using Gladiator and Horizon rotation rate sensors, the first two rotational modes were obtained. The rotational modes from the direct measurements were compared with the rotational modes derived from the acceleration measurement’s central difference approximation and theoretical FEM results. Both direct and indirect results were compatible with the FEM theoretical first rotational mode using MAC values >0.990. By using rotation rate sensors, one could get better information about the first rotational mode in a certain area of the beam, i.e., at the end and in the middle. For the second natural mode, the direct rotational mode was in less agreement with the rotational mode from the central difference approximation (MAC values 0.833–0.972). It can be explained by the second natural frequency exceeding the bandwidth of the sensor used in the experiment, and not enough measurement points for translational measurements to calculate the accurate mode derivative.

Rotational modes were also obtained for the asymmetrically damaged beams. Inflicted damage resulted in a decrease in the first natural frequencies by 12.9–17.5%. As could be expected, the damage inflicted to the beams influenced the rotational natural modes to a greater extent (MAC values <0.9854) than the translational natural modes (MAC values < 0.9947).

Applying rotation rate sensors, the rotational deflection shape tests were obtained from excitations induced by an inertial vibration exciter (Tira). The obtained deflection shapes are compatible with the rotational modes obtained from the impact hammer tests.

Following previous experience on small plexiglass beams [4], the two rotation rate sensors were also used to measure the strain rate for the UHPC beams, spatially averaged at a 0.75 m distance. Despite the different scale of this experiment, we were able to acquire the strain rates measured on real-sized UHPC beams during harmonic excitations. The obtained, steady-state strain rates were equal to ${\dot{\varepsilon }}_{max}=5\times 10^{-3}\;s^{-1}$, while the steady-state strains were equal to $\varepsilon =3\times 10^{-5}$. The experiments demonstrated that the measured strain rates could also be used to control the strains during dynamic tests. It may be useful to limit unwanted cracks in the elements in any type of experiments involving concrete specimens.

The rotation rate sensors can be used in the modal analyses of full-scale civil engineering structures. When more effective and cheaper devices are developed, rotation rate sensors may be employed in the everyday practice of SHM in civil engineering.

## Figures and Tables

**Table 1:** Basic mechanical properties of the beams.

Beam Symbol	Fibre Type	Density (kg/m^3^)	Compressive Strength (MPa)	Tensile Strength in Flexure (MPa)
UHPC1_L	Glass	2 301	122	8.60
UHPC2_L	Steel	2 625	238	10.13

**Table 2:** Sensor properties.

Sensor Name	Type	Measurement Range	Bandwidth	Resolution
Horizon HZ1-100-100	MEMS	±100 deg/s	60 Hz	0.0005°/s
Gladiator G150z	MEMS	±100 deg/s	200 Hz	0.004°/s

**Table 3:** Sensor position.

Axis		1	2	3	4	5	6	7	8	9
**Sensor Type**	UHPC1_L	G	G	G	H	H	H	G	G	G
	UHPC2_L	H	G	G	G	H	G	G	G	H

G—Gladiator; H—Horizon rotation rate sensor.

**Table 4:** Modal parameters for the first three modes (least squares complex exponential (LSCE) method) in the intact state of the beams.

Mode	Beam UHPC1_L		Beam UHPC2_L	
	f_n_ [Hz]	ξ [%]	f_n_ [Hz]	ξ [%]
1	23.51	0.555	27.06	0.345
2	64.52	0.511	74.18	0.504
3	127.33	0.577	140.66	0.818

**Table 5:** MAC values for the first rotational mode for the UHPC1_L beam. Intact state.

	Direct Hammer	Direct FEM	Derivative Hammer	Derivative FEM
Direct Hammer	1.000	0.999	0.998	0.997
Direct FEM	0.999	1.000	0.999	0.999
Derivative Hammer	0.998	0.999	1.000	1.000
Derivative FEM	0.997	0.999	1.000	1.000

**Table 6:** MAC values for the first rotational mode for the UHPC2_L beam. Intact state.

	Direct Hammer	Direct FEM	Derivative Hammer	Derivative FEM
Direct Hammer	1.000	0.996	0.995	0.994
Direct FEM	0.996	1.000	0.999	0.999
Derivative Hammer	0.995	0.999	1.000	1.000
Derivative FEM	0.994	0.999	1.000	1.000

**Table 7:** MAC values for the second rotational mode for the UHPC1_L beam. Intact state.

	Direct Hammer	Direct FEM	Derivative Hammer	Derivative FEM
Direct Hammer	1.000	0.991	0.972	0.986
Direct FEM	0.991	1.000	0.975	0.986
Derivative Hammer	0.972	0.975	1.000	0.995
Derivative FEM	0.986	0.986	0.995	1.000

**Table 8:** MAC values for the second rotational mode for the UHPC2_L beam. Intact state.

	Direct Hammer	Direct FEM	Derivative Hammer	Derivative FEM
Direct Hammer	1.000	0.944	0.833	0.898
Direct FEM	0.944	1.000	0.947	0.986
Derivative Hammer	0.833	0.947	1.000	0.986
Derivative FEM	0.898	0.986	0.986	1.000

**Table 9:** Modal parameters for the first three modes (LSCE method) in the damaged state of the beams.

Mode	Beam UHPC1_L		Beam UHPC2_L	
	f_n_ [Hz]	ξ [%]	f_n_ [Hz]	ξ [%]
1	19.40	1.78	23.56	0.883
2	55.44	1.41	65.11	1.318
3	111.08	1.71	131.45	0.793
